# Supplementary material for: Advances in the Design and Functionality of a Compact Multi‐Reflecting Time‐of‐Flight Mass Spectrometer
Source: Rapid Commun Mass Spectrom. 2026 Sep 9;40(22):e70173. doi: 10.1002/rcm.70173 (PMC13556519; doi:10.1002/rcm.70173)
Supplement: Supplementary file 1 [file RCM-40-e70173-s001.docx]

**Supporting Information**

**Advances in the Design and Functionality of a**

**Compact Multi-Reflecting Time-of-Flight Mass Spectrometer**

Jason Wildgoose ^1^, Samantha Ferries ^1^, Lee A. Gethings ^1^ , Matthew E. Daly ^1^,

Martin E. Palmer ^1^, Richard Lock ^1^, Johannes P.C. Vissers ^1^, James I. Langridge ^1,^*

1.	Waters Corporation, Altrincham Road, Wilmslow, SK9 4AX, United Kingdom

*	[james_langridge@waters.com](mailto:james_langridge@waters.com)

**Table S1.** Infusion MS resolution measurements (n = 6) for solvent standards.

|  |  | acquisition rate (Hz) | | | | |
| --- | --- | --- | --- | --- | --- | --- |
| compound | *m/z* (Da) | 10 | 20 | 50 | 100 | 200 |
| 15:0-18:1(d7) DAG  15:0-18:1(d7) PC  15:0-18:1(d7) PE  15:0-18:1(d7)-15:0 TAG  18:1(d7) Lyso PC  18:1(d7) Lyso PE  C15 Ceramide-d7  d18:1-18:1(d9) SM | 605.5844  753.6134  711.5664  829.7985  529.3994  487.3524  531.5477  738.6470 | 55,336  58,284  53,041  52,181  58,840  52,319  52,177  51,615 | 54,465  57,397  51,783  48,789  56,467  51,616  50,461  51,236 | 55,977  58,017  53,223  50,999  57,604  53,223  53,950  52,392 | 61,481  58,031  53,189  52,400  55,463  56,881  53,951  56,471 | --  --  --  --  --  --  --  -- |

**Table S2.** Infusion MS/MS resolution measurements (n = 6) for solvent standards (precursor *m/z* > precursor *m/z*).

|  |  | acquisition rate (Hz) | | | | |
| --- | --- | --- | --- | --- | --- | --- |
| compound | *m/z* (Da) | 10 | 20 | 50 | 100 | 200 |
| 18:1(d7) Lyso PC | 529.3994 | 50,340 | 49,169 | 48,354 | 49,501 | 48,027 |

**Table S3.** Infusion MS/MS resolution measurements (n = 6) for solvent standards (precursor *m/z* > product *m/z*).

|  |  | acquisition rate (Hz) | | | | |
| --- | --- | --- | --- | --- | --- | --- |
| compound | *m/z* (Da) | 10 | 20 | 50 | 100 | 200 |
| 18:1(d7) Lyso PC | 184.0733 | 45,931 | 44,651 | 45,311 | 42,053 | 40,199 |

**Table S4.** Theoretically predicted and experimentally detected fragment ion *m/z* values for TG 15:0/16:1/18:0.

| elemental composition fragment ion *** | adduct | theoretical *m/z* ^Δ^ | observed *m/z* ^‡^ | error (ppm) | comment |
| --- | --- | --- | --- | --- | --- |
| C_9_H_15_  C_14_H_29_  C_15_H_29_O  C_16_H_31_O  C_18_H_33_O  C_18_H_35_O_2_  C_36_H_67_O_4_  C_52_H_97_O_5_  C_52_H_99_O_6_ | [M+H]+  [M+H]+  [M+H]+  [M+H]+  [M+H]+  [M+H]+  [M+H]+  [M+H]+  [M+H]+ | 123.11737  197.22691  225.22183  239.23748  265.25313  283.26369  563.50391  801.73356  819.74413 | 123.1174  --  225.2215  239.2369  265.2530  --  563.5037  --  819.7410 | 0.3  --  -1.5  -2.4  -0.5  --  -0.4  --  -3.8 | low abundant  low abundant  low abundant  low abundant |

* <http://cfmid4.wishartlab.com/>

^Δ^ <https://pnnl-comp-mass-spec.github.io/Molecular-Weight-Calculator-VB6/>

^‡^ <https://systemsomicslab.github.io/compms/msdial/main.html>

**Figure S1.** Summed detail infusion MS/MS spectra acquired following precursor ion isolation at *m/z* 529.3994 with CID (*m/z* 529.3994 > *m/z* 184.0733), collected at acquisition rates ranging from 10 to 200 Hz. FWHM resolution as a function of MS/MS acquisition rate for 18:1(d7) Lyso PC (*n* = 6). Error bars represent two standard deviations.

**Figure S2.** Normalized (FHWH_10 Hz_ = 1) average FWHM resolution (n = 6) as a function of LC-MS and LC-MS/MS acquisition rate in the absence (top row) and presence (bottom row) of urine matrix. Blue = 15:0-18:1(d7) DAG; orange = 15:0-18:1(d7) PC; green = 15:0-18:1(d7) PE; red = 15:0-18:1(d7)-15:0 TAG; purple = 18:1(d7) Lyso PC; brown = 18:1(d7) Lyso PE; pink = C15 Ceramide-d7; grey = d18:1-18:1(d9) SM. The LC-MS/MS matrix data were evaluated using the A+1 isotope peak.

**Figure S3**. Base Peak Intensity (BPI) and precursor MS1 Extracted Ion Chromatogram (EIC), shown inset, broadband DIA (MSE) chromatograms of dihydrocodeine (1, green), codeine (2, orange), oxycodone (3, purple), 6-acetylmorphine (4, blue), and fentanyl (5, red), spiked at about 40 ng/mL into urine matrix.

**Figure S4**. MS1 and MS2 Extracted Ion Chromatogram(s) (EIC) 6-acetylmorphine (top) and dihydrocodeine (bottom) spiked in urine matrix at 4.57 (left) and 370.37 (right) ng/mL.

**Figure S5.** Example stepped quadrupole DIA Base Peak Intensity (BPI) chromatograms of (A) bipolar, (B) schizophrenia, (C) control, and (D) study pool QC lipid extract plasma samples from a mental disorder study using fast, narrow‑bore LC separations.

**Figure S6**. (A) Base‑peak intensity (BPI) chromatogram and annotated raw MS2 product ion spectra showing *y*‑ and *b*‑ions for (B) high‑abundance (IAAANVPAFVSGK | P0ACF0 – DBHA_ECOLI | 7.24 min), (C) mid‑abundance (GQNVPVVFFNK | P0AEE5 – MGLB_ECOLI | 8.09 min), and (D) low‑abundance (LTGLEGEQLGIVSLR | P0A707 – IF3_ECOLI | 9.38 min) peptides acquired using stepped‑quadrupole DIA with a 20 min gradient (inject‑to‑inject cycle time). Data were obtained from 4 µg of an *E. coli* tryptic digest loaded on‑column, spanning at least two orders of magnitude in summed product ion area. Across the experiments, the root mean square (RMS) of the mean precursor and product ion mass errors was 0.47 and 2.45 ppm, respectively. The corresponding mean within-experiment standard deviations were 2.5 and 6.0 ppm.

**Figure S7.** (A) Protein groups identified in an *E. coli* tryptic digest, (B) protein groups quantified with coefficients of variation (CV) below 10 % (dark blue), between 10 % and 20 % (mid blue grey), and above 20 % (light blue grey), and (C) protein group intersection for different on‑column amounts and chromatographic gradient lengths (technical replicates, n = 3), determined using an individual‑run‑based analysis approach.

**Figure S8.** (A) Peptides detected in an *E. coli* tryptic digest, (B) peptides quantified with coefficients of variation (CV) below 10 % (dark blue), between 10 % and 20 % (mid blue), and above 20 % (light blue) and (C) peptide intersection for different on‑column amounts and chromatographic gradient lengths (technical replicates, n = 3), determined using an individual‑run‑based analysis approach.
